# Lead Levels in a Potters Population and Its Association With the Use of Different Glazes: Cross-Sectional Evaluation of the *Approved Pottery Program*

**DOI:** 10.3389/ftox.2022.799633

**Published:** 2022-03-07

**Authors:** Netzy Peralta, Alejandra Cantoral, Martha María Téllez-Rojo, Belem Trejo-Valdivia, Daniel Estrada-Sánchez, Vesta Richardson-L, Jack Caravanos, Richard Fuller

**Affiliations:** ^1^ Institute for Research in Humanities and Social Sciences, Universidad Autónoma Del Estado de Morelos, Cuernavaca, Mexico; ^2^ National Institute of Public Health (Mexico), Cuernavaca, Mexico; ^3^ Pure Earth, New York, NY, United States; ^4^ Mexican Social Security Institute (IMSS), Mexico City, Mexico; ^5^ School of Global Public Health, New York University, New York City, NY, United States

**Keywords:** blood lead levels, pottery, lead-free glaze, artisans, soil lead levels

## Abstract

Lead is one of the most harmful toxic metals to humans. In Mexico, though most potters still use a lead-based glazing process, a new lead-free glaze has been introduced to the production of pottery. The Approved Pottery Program (APP) promotes the production of lead-free pottery. As a component of the APP, we aimed to document in this pilot study the blood lead levels (BLLs) of a sample of potters and the association with the type of glaze used. A cross-sectional study was conducted based on information from 46 potters grouped by 26 workshops. We measured general sociodemographic characteristics, capillary BLLs, and the lead levels of the dirt floors of the workshops. The evaluation of associations and comparisons between glaze types was performed based on a regression model clustered by workshop. The median BLL measured was 13.6 μg/dl (IQR: 7.8–20.4 μg/dl), and 70% of the BLLs were greater than 10 μg/dl. Workshop managers presented higher BLLs compared to others working in the same workshop (median of 14.1 μg/dl (IQR: 11.6–25.3 μg/dl) versus 10.1 μg/dl (IQR: 5.2–16.7 μg/dl), respectively). The median BLLs of potters who used lead-free glaze in at least 80% of production were 8.8 μg/dl (95% CI: −17.3 to −0.3 μg/dl) lower than the BLLs of those who used lead-free glaze in less than 30% of production, adjusted by workshop role. Additionally, the lead levels were significantly lower in workshop dirt floors where lead-free glaze was used in at least 80% of the production compared to those that use less than 30% (180 versus 916 mg/kg; *p* < 0.05). The use of lead-free glaze in the production of pottery was associated with both lower BLLs in potters and lower soil lead levels in the workshop area.

## Introduction

Lead is a highly toxic heavy metal (NRC, 1993). After entering the human body, it targets multiple organs, including the brain, kidneys, liver, and bones (Silbergeld et al., 1993), causing chronic intoxication. As a result, the World Health Organization (WHO) considers lead one of ten chemicals of greatest concern for public health (WHO, 2010). Although blood lead levels (BLLs) in the Mexican population have declined significantly in the last 25 years following the elimination of lead in gasoline (Pantic et al., 2018; Rothenberg et al., 1998), toxic exposure persists in the general population due to the presence of lead in food and beverages cooked or stored in low-temperature glazed pottery (LTGP) (Rojas-Lopez et al., 1994; Romieu et al., 1994; Tellez-Rojo et al., 2017; Tellez-Rojo et al., 2019), despite a national law that has established lead limits in pottery (NOM-011-SSA1, 1993).

For over four decades, studies have demonstrated that chronic lead exposure causes health problems; namely, hypertension at levels of 10 μg/dl (Kim et al., 2019; Nawrot, et al., 2002), adverse renal effects at levels of 30 μg/dl (Diamond, 2005), and systemic poisoning (causing dementia, irreversible damage to organs and systems, and other effects) at concentrations above 60 μg/dl (ATSDR, 2007). Furthermore, even at low levels (6.7 μg/dl), lead exposure has been documented as a risk factor for cardiovascular disease mortality (Lanphear et al., 2018; NTP, 2012;, 2018). The reference in Mexico for lead levels in an occupationally exposed population, which includes potters, is 10 μg/dl in whole blood for women (regardless of pregnancy status) and 30 μg/dl for men (NOM-047-SSA1, 2011).

Although the National Fund for the Promotion of Handicrafts (FONART) in Mexico has introduced the use of lead-free glazes through the project Strategic Program for the Replacement of Lead in Traditional Glassware Pottery (1994), these glazes have not been widely adopted because they do not have the fusion or aesthetic characteristics of lead oxide. In late 2010, boron-based enamels were introduced, which can be used in traditional kilns and have aesthetic characteristics similar to those of lead oxide. Nevertheless, potters continue to use the traditional glaze method, despite significant efforts by various non-government organizations and government institutions to eliminate its use.

### Approved Pottery Program (Programa Barro Aprobado, APP)

Pottery is a traditional livelihood for at least 10,000 artisans in 20 Mexican states. Morelos in central Mexico is one such lead-glazed pottery producing states. Most producers in Morelos reside in the community of Tlayacapan and have been producing traditional pottery for generations. In Tlayacapan, a total of 60 pottery workshops producing mainly LTGP ceramics are registered. Potters share workspaces around one kiln used to melt ceramic pieces; in this sense, the 60 workshops are clustered into 21 workspaces called domestic units.

APP, initiated by the international environmental health organization Pure Earth (formerly known as Blacksmith Institute) in 2014, is a multi-institutional project that promotes the production of lead-free LTGP (Barro-Aprobado, 2014). The objective of APP is to eliminate the use of lead oxide in pottery by educating potters and encouraging demand for lead-free LTGP among consumers, including restaurants and other vendors, so as to decrease the primary source of non-occupational exposure to lead in Mexico. The APP strategy seeks to accomplish this objective through the following activities: train potters in the use of lead-free glaze, remediate the contaminated workshops of potters who have agreed to stop using lead oxide, and monitor the BLLs of potters and relatives who assist in the production process. Monitoring lead exposure in a population is a complex task, requiring the identification of suitable and accessible biomarkers to implement prevention programs, management, and public health decisions (Barbosa et al., 2005; Sanders et al., 2009).

The primary objective of this study was to document the BLLs of a sample of adult potters from Tlayacapan County, Morelos, in central Mexico, to analyze how lead levels vary according to the use of lead-based versus lead-free glaze.

## Materials and Methods

This research consisted of a pilot study with a cross-sectional design conducted between January to March of 2015. Of 60 total registered pottery workshops producing ceramics in Tlayacapan in 2015, 30 were operated throughout the year while the remainder were operated only seasonally (when the demand for ceramics increased due to religious festivities). The research staff contacted workers (the manager and relatives working in the workshop) from each workshop and explained the objective of the project. Those that agreed to participate signed an informed consent letter. The analytical sample included 46 participants (workshop managers and their relatives, including women) whom completed the questionnaires from 26 workshops.

The health professionals followed a standardized protocol to measure the concentrations of lead in capillary blood of at least one family member (including the workshop manager and relatives who participated in the production process), a total of 43 participants accept the capillary blood test. A LeadCare II device (Model: LeadCare Analyzer, ESA Inc., Serial Number: LC1722) was used for the procedure. This test presented an overall sensitivity of 71.8% and a positive predictive value of 76.3% (Gonzalez et al., 2019). The fingertip was thoroughly wiped using an alcohol swab, and upon lancing, a total of 50 μl of capillary blood was taken. The capillary blood was then mixed with an equal amount of anticoagulant and placed on an electrochemical sensor strip. The resultant reading was generated in 180 s.

The manager of each workshop completed a general questionnaire about their knowledge of the health impacts of lead exposure, alternatives to the use of lead-free glaze, reasons for continuing to use the glaze, and estimated percentage of lead-based glaze versus lead-free glaze LTGP production in the workshop (because many potters use both glazes during production).

Additionally, we performed a workshop environment evaluation. The lead levels in soil were measured using a portable direct reading X-ray fluorescence analyzer (Model: XRF, Alpha 6,500 from Innov-X Systems, Serial Number: 9,162). This device detects approximately two dozen elements in a non-destructive manner with very high precision (Shefsky, 1997). Specifically, collected samples achieve a sensitivity of 87%, a specificity of 98%, and an accuracy of 96% for detection of environmental lead hazards (Tighe et al., 2020). Readings were taken in the workshops and other areas where workers apply the lead-based or lead-free glaze. A minimum of four measurements testing dirt or dusty surfaces were taken in each workshop. For this analysis, only measurements close to the kiln (within a 1 m radius) were included.

This study was approved by the Ethics Commission of the Health Services of Morelos and the City University of New York.

### Statistical Analysis

Due to the asymmetric behavior of the distribution of lead levels in blood (bias to the right), the descriptions of lead levels are presented as median and interquartile ranges. Given the main aim of this analysis was to compare lead levels according to the use of lead-based versus lead-free glaze in the production of LTGP, and based on information reported by the workshop managers, the percentage of lead-free glaze used in the workshop production was categorized as follows: low (<30%), medium (30–80%), and high (>80%). This categorization was performed in order to include the most frequent answers given by the potters when asked about the use of lead-free glaze (responses were: none or less than a third; or almost always).

We used non-parametric Kruskal-Wallis tests for comparisons between groups: percentage of use of lead-free enamel (low, medium, high); workshop role (managers vs. their relatives working under the same conditions); and sex (men vs. women). Soil lead levels were also compared through the Kruskal-Wallis test according to the use of lead-free enamel (low, medium, high). We estimated a correlation between blood and soil lead levels through a regression model that considered the clustering of workshops (as the 43 participants were clustered in 26 workshops).

Evaluation of the association between the percentage of lead-free glaze use (low, medium, high) and lead levels in capillary blood was based on a linear regression model for grouped data (workshops) and adjusted by occupational role (manager vs. other relatives). All analyses were done using Stata 15 software (StataCorp, 2017).

## Results

A description of the 46 potters clustered by workshop can be found in Table 1. The number of participants range from 1–6, and in the case of workshops with only one potter, the potter included was the manager of the workshop (Table 1).

**TABLE 1:** Detailed description of the workshops.

Workshop ID	Number of potters	Sex (% male)	Mean age (years) Mean (SD)	Lead in capillary blood (μg/dl)		Lead-free glaze used in production
				Median	IQR	%
1	1	100	67	18.8		0
2	1	100	43	12.6		0
3	1	100	24	N/A		0
4	1	100	67	25.3		0
5	1	100	52	18		0
6	3	66.6	73.6 (13.05)	17.6	14.5–21.8	0
7	2	100	34.5 (19.1)	11.8	8.6–14.9	0
8	1	100	54	4.4		0
9	1	100	67	10.2		0
10	1	100	63	13.5		0
11	1	100	43	6		0
12	2	100	33.5 (20.5)	46.5	27.9–65	10
13	1	100	62	65		20
14	2	50	43.3 (18.17)	21.9	7.1–36.6	20
15	1	100	40	11.6		30
16	2	50	35.3 (3.5)	10.4	10.2–10.6	40
17	3	66.6	50 (10.8)	18.6	16.8–65	50
18	1	100	41	N/A		50
19	1	100	26	24.2		50
20	2	100	44.5 (20.5)	29	20.4–37.5	50
21	1	100	53	N/A		50
22	2	50	48.3 (19.5)	14.3	7.8–20.7	50
23	2	50	31 (7.07)	9.6	5.5–13.7	50
24	2	50	59 (4.2)	12.6	11.7–13.4	90
25	6	66.6	35.5 (13.7)	5.8	3.8–14.1	100
26	4	25	55.2 (21.1)	7.3	4.0–11.6	100
***Total***	***46***		***47.2*** (***16.8***)	***13.6***	***7.8***–***20.4***	

According to the general questionnaire distributed to the manager of each workshop, the median age of the potters was 50 years (mean: 47.2 ± 16.8 years) with a range of 19–84 years, with no difference between groups in the percentage of lead-free glaze use in the workshop. The majority of participants were men (62.5%). More than 80% of workshops reported frequent use of lead glaze in their production processes and having used lead glaze for more than 25 years. The main reason cited for continuing the use of lead glaze was demand from clients to obtain a shinier product (23.1%). Sixty-one percent of potters reported being aware of the availability of lead-free glaze; however, these potters only used lead-free glaze in less than 30% of their production process. Only two workshops participated in the APP; as a result, their ceramic production was 100% lead-free. Additionally, one workshop used 90% lead-free glaze. One of these three lead-free or nearly lead-free workshops was led by a woman, which is contrary to the norm of male leaders in pottery communities. Almost half of the potters (46%) acknowledged and recognized the adverse health effects of lead exposure.

**FIGURE 1:**
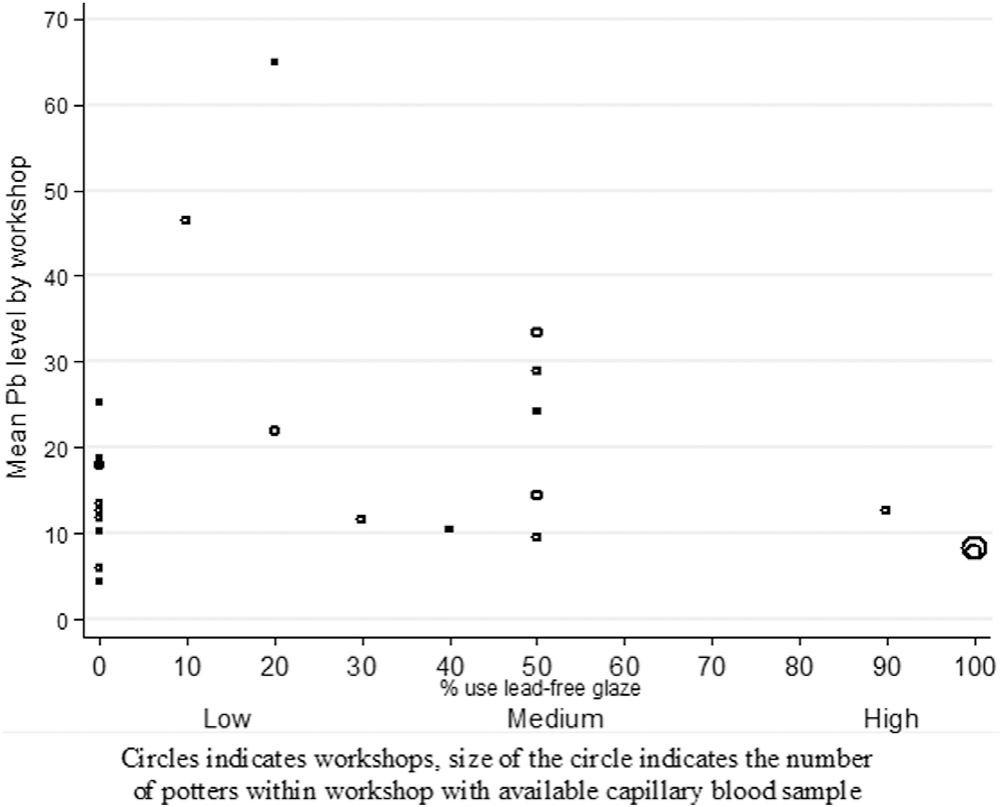
Capillary blood lead levels (mcg/dl) cluster by workshop and presented by use of lead-free glaze.

According to the percentage of lead-free glaze use, the 26 workshops were distributed as follows: 13 had low use of lead-free glaze in the LTGP production (<30%), 10 had medium use (30–80%), and only three had high use (>80%). The number of potters per workshop ranged from 1–6 for a total of 43 individual blood samples for the 26 workshops (Figure 1). The median BLL was 13.6 μg/dl (IQR: 7.8–20.4 μg/dl) with a range of 3.2–65 μg/dl. None of the participants had values below LeadCare’s 3.3 μg/dl limit of detection, and two potters presented the highest value detected (65 μg/dl). There was a significant difference (*p* < 0.01) in the median BLLs when compared by percentage of lead-free glaze use, with a median value of 8.1 μg/dl (IQR 4.35–13.5 μg/dl) for those in the high use category, 14.9 μg/dl (IQR: 10.6–20.4 μg/dl) for medium use, and 17.8 μg/dl (IQR: 11.2–23.3 μg/dl) for low use (Table 2).

**TABLE 2:** Levels of lead in capillary blood (μg/dl) by workshop and use of lead-free glaze.

Percentage of lead-free glaze use in the production	Workshops	Potters	Lead levels (μg/dl)		p*
%	N (%)	N (%)	Median	IQR	
<30	13 (50)	18 (42)	17.8	11.2–23.3	0.008
30–80	10 (30)	13 (30)	14.9	10.6–20.4	
>80	3 (12)	12 (28)	8.1	4.35–13.5	
Total	26 (100)	43 (100)	13.6	7.8–20.4	

* *p*-value: Kruskal-Wallis test for comparison among the three groups

Soil lead levels, which were only measured in eight workshops, presented a median value of 195 mg/kg (IQR: 165–916 mg/kg) around the kiln. Values were lower in the workshops that used >80% of lead-free glaze (180 mg/kg; IQR: 165–195 mg/kg) compared to the medium and low use categories (*p* < 0.05) (Table 3).

**TABLE 3:** Soil lead levels (mg/kg) by workshop and use of lead-free glaze.

Percentage of lead-free glaze use in the production	Workshops	Lead levels (mg/kg)		p*
%	N	Median	IQR	
<30	2	916	916–1702	0.006
30–80	3	916	458–2,422	
>80	3	180	165–195	
Total	8	195	165–916	

* *p*-value: Kruskal Wallis test for comparison among the three groups

**FIGURE 2:**
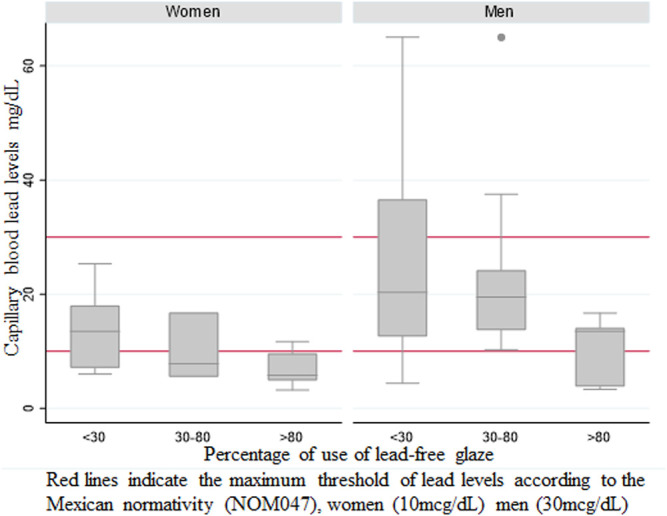
Capillary blood lead levels (mcg/dl) by sex and according to the declared percentage of use of lead-free glaze.

The median BLL of managers was 14.1 μg/dl (IQR: 11.6–25.3 μg/dl), which was higher than the median BLL of the relatives who assist in the workshop (median: 10.1 μg/dl; IQR: 5.2–16.7 μg/dl). Additionally, men presented higher levels than women, independent of their role in the workshop (median: 15.8 μg/dl; IQR: 12.6–24.2 μg/dl and median: 9.6 μg/dl; IQR: 6–13.5 μg/dl, respectively). Figure 2 presents the variation of BLLs according to the percentage of lead-free glaze use stratified by gender. This figure shows that 71% of participants had lead levels ≥10 μg/dl. Furthermore, 47% of women had levels above 10 μg/dl, and 19% of men had levels above 30 μg/dl, which exceeds the standard reference values for occupationally exposed workers (NOM-047-SSA1, 2011).

The adjusted model estimated that potters who used <30% of lead-free glaze in the production of LTGP had BLLs an average of 8.8 μg/dl (95% CI: 0.3–17.3 μg/dl) higher than potters who use >80% of lead-free glaze in their production (Table 4).

**TABLE 4:** Association of lead levels in capillary blood (μg/dl) according to the use of lead-free glaze in production and pottery (N = 43), grouped into 26 workshops (cluster).

	Coefficient	95% CI	
Percentage of lead-free glaze use in the production			
<30% (N = 18)	reference		
30–80% (N = 13)	−0.37	−14.5	13.7
>80% (N = 12)	−**8.8**	−**17.3**	−**0.3**
Manager of the workshop			
No (N = 20)	reference		
Yes (N = 23)	**8.9**	**2.4**	**15.4**

Bold values indicate *p* value < 0.05

## Discussion

This pilot study describes one of the most lead-exposed populations in Mexico. The results show high BLLs in the evaluated population as well as how the APP efforts to replace lead glaze correlate with BLLs. The median capillary BLLs in the potters that produce LTGP (occupationally exposed sample) in Tlayacapan County, Morelos, was 13.6 μg/dl (IQR: 7.8–20.4 μg/dl), with a maximum value of 65 μg/dl (in two participants). The proportion of potters with BLLs above the action level established by Mexican regulations for occupationally exposed populations was 19% in men and 47% in women. This finding is alarming, given that numerous international agencies and studies suggest that there are adverse health effects, such as hypertension, even at levels below 30 μg/dl (Nawrot et al., 2002; NTP, 2012). Previous studies have also identified Mexican potters as being heavily exposed to lead-based glaze (Diaz-Ruiz et al., 2017; Tamayo-Ortiz and Navia-Antezana, 2018).

Similar to our results, previous studies on lead monitoring in occupationally exposed workers reported BLLs between four to seven times higher than in controls (not occupationally exposed) and higher BLLs in men than women (Kasuba et al., 2020; Shraideh et al., 2018). Another study on occupational lead exposure noted that 71% of employes at a lead oxide manufacturing presented BLLs >30 μg/dl (Rinsky et al., 2018).

This study also reveals that most potters report knowing about the existence of lead-free glaze while acknowledging the adverse effects of lead on health. Nonetheless, the majority continue to use lead oxide in the glaze, primarily because consumers prefer this method, as it is thought to impart a shinier and more polished (professional) surface. These findings reflect a lack of awareness among consumers (including the general population and restaurant owners) and suggest that, if demand for lead-free glazed pottery were increased, potters would be incentivized to stop using lead-based glaze, resulting in a decrease in the health risks to potters and their families. Currently, outlets that sell 100% lead-free glaze pottery are scarce (FONART, 2010).

The reported percentage of lead-free glaze used in production has an inverse association with capillary BLLs; a higher percentage of lead-free glaze used in production is associated with lower BLLs on average among its users. Potters with at least 80% lead-free glaze production have almost 10 μg/dl lower capillary BLLs than those with less than 30% of lead-free glaze production. These results convincingly demonstrate the efficacy of lead-free training programs.

Because workshops are organized in family units to fire the ceramic pieces, this may represent a cross-contamination source, given that the same kilns are used by two to three workshops. As a result, even if a family produces completely lead-free pottery, their production may be contaminated with lead during the firing process in public kilns. Although a previous study in the state of Hidalgo suggests that clay pieces with lead-based glaze do not contaminate unleaded pottery burnt in the same kiln (COPRISEH, 2011), it may be worth conducting a follow-up study to explore this behavior.

Workshops that used mostly lead-free glaze had lower average levels of lead in soil around the kiln. It is important to note that half of the workshops studied registered values above 400 mg/kg of lead, and two workshops registered values above 1,000 mg/kg (NOM-147-SEMARNAT-SSA1, 2004). This result is consistent with a previous analysis of seven states in Mexico, where authors found a mean lead concentration above 1,000 mg/kg (Estrada-Sanchez et al., 2017). Decision-makers and authorities should consider this finding and support lead-free potters, as well as provide new kilns or manage a cleaning program within the workshops.

Our study has several limitations. First, while measuring venous whole blood is an accurate method for gauging lead exposure, its invasive nature of venipuncture may pose challenges, such as the need for clinical settings, well-trained personnel, specialized materials, and specific conditions for sample transportation and storage (Barbosa et al., 2005; Nyanza et al., 2019). These challenges can be addressed by employing alternative methods, such as capillary blood sampling (Tang et al., 2017), and studies have reported relatively high correlation in lead measures between the two sources of blood from a given individual (Resano et al., 1994;, 2007). Another limitation is that the equipment used to measure capillary BLLs, Lead Care, has a limit of detection (LOD) of 3.3 μg/dl. While this means that levels below the LOD will be excluded, the capillary samples in this study all measured above the mentioned LOD. Furthermore, the sample population from this study may not be representative of the entire potter population in the Tlayacapan County, due to the self-selected sampling of workshops and participants. In the case of the workshop managers who did not agree to participate, no information could be obtained that would allow comparison with participants. However, prior observation in the community suggests that families refusing to participate tend to use lead glaze in production, meaning that the contamination and exposure levels found in this population are potentially underestimates. Finally, although many workshop managers answered the questionnaire, some did not give consent for blood sampling, which also reduced the sample size for the biomarker analysis.

Training efforts on the use of lead-free glaze in pottery production show positive effects on population health. Nonetheless, efforts to incentivize potters to adopt the lead-free method should be expanded to reduce BLLs in the occupationally exposed population. Additionally, the regulations that limit lead in pottery (NOM-231-SSA1, 2002) should be enforced by state authorities belonging to the Federal Commission for the Protection Against Sanitary Risks (COFEPRIS), the entity responsible for verifying the commercialization sources of LTGP. Nonetheless, the nature of LTGP production, sale, and distribution make the monitoring and enforcement of this law difficult, given that potters often have domestic workshops yet sell their pottery nationally rather than solely in their municipality. One recent step taken to reduce exposure due to lead-based glaze is the creation by the Ministry of Health of the Program for Immediate Action in the Control of Lead Exposure (Salud, 2019), which is a multisectoral program to reduce all sources of lead exposure, including lead-based glaze in LTGP.

Because this was a pilot study, more research with occupationally exposed populations, such as artisans, should be conducted in other areas of Mexico to confirm the results of this analysis.

## Data Availability

The original contributions presented in the study are included in the article/Supplementary Material, further inquiries can be directed to the corresponding author.

## Abbreviations

APP, Approved Pottery Program; BLLs, Blood lead levels; LTGP, Low-temperature glazed pottery; FONART, National Fund for the Promotion of Handicrafts (Fondo Nacional para el Fomento de las Artesanías); WHO, World Health Organization.

## References

[B1] Atsdr (2007). Toxicological Profile for lead. Atlanta, GA: U.S. Department of Health and Human Services Public Health Service Agency for Toxic Substances and Disease Registry.

[B2] Barbosa F. Jr. Tanus-Santos J. E. Gerlach R. F. Parsons P. J. (2005). A Critical Review of Biomarkers Used for Monitoring Human Exposure to lead: Advantages, Limitations, and Future Needs. Environ. Health Perspect. 113 (12), 1669–1674. 10.1289/ehp.7917 16330345 PMC1314903

[B3] Barro-Aprobado (2014). Program by Pure Earth. Ciudad de México, Mexico: Barro Aprobado. Available at https://barroaprobado.org/.

[B4] Copriseh (2011). Pruebas de Plomo a Piezas de Alfaería con Vidriado Libre de Plomo Quemadas en Horno Tradicional Sin Remediar. Retrieved from: http://alfareria.org/sites/default/files/Pruebas.pdf.

[B5] Diamond G. L. (2005). “Risk Assessment of Nephrotoxic Metals,” in The Toxicology of the Kidney. (London: CRC Press), 1099–1132.

[B6] Diaz-Ruiz A. Tristán-López L. A. Medrano-Gómez K. I. Torres-Domínguez J. A. Ríos C. Montes S. (2017). Glazed clay Pottery and lead Exposure in Mexico: Current Experimental Evidence. Nutr. Neurosci. 20 (9), 513–518. 10.1080/1028415X.2016.1193967 27297776

[B7] Estrada-Sánchez D. Ericson B. Juárez-Pérez C. A. Aguilar-Madrid G. Hernández L. Gualtero S. (2017). Intelligence Quotient Loss in Mexican Pottery Artisan's Children. Rev. Med. Inst. Mex Seguro Soc. 55 (3), 292 28440982

[B8] FONART (2010). Uso de plomo en la Alfarería en México, Informe 2010. Available at: http://alfareria.org/sites/default/files/images/InformePbAlfareria2010.pdf .

[B9] González F. Camacho M. Tiburón N. P. Peña M. Z. Rueda L. R. Luzardo O. P. (2019). Suitability of Anodic Stripping Voltammetry for Routine Analysis of Venous Blood from Raptors. Environ. Toxicol. Chem. 38 (4), 737–747. 10.1002/etc.4339 30556155

[B10] Kašuba V. Milić M. Želježić D. Mladinić M. Pizent A. Kljaković-Gašpić Z. (2020). Biomonitoring Findings for Occupational lead Exposure in Battery and Ceramic Tile Workers Using Biochemical Markers, Alkaline Comet Assay, and Micronucleus Test Coupled with Fluorescence *In Situ* Hybridisation. Arh Hig Rada Toksikol 71 (4), 339–352. 10.2478/aiht-2020-71-3427 33410779 PMC7968510

[B11] Kim M. G. Kim Y. W. Ahn Y. S. (2019). Does Low lead Exposure Affect Blood Pressure and Hypertension? Jrnl Occup. Health 62. 10.1002/1348-9585.12107 PMC6970393 31858671

[B12] Lanphear B. P. Rauch S. Auinger P. Allen R. W. Hornung R. W. (2018). Low-level lead Exposure and Mortality in US Adults: a Population-Based Cohort Study. The Lancet Public Health 3 (4), e177–e184. 10.1016/S2468-2667(18)30025-2 29544878

[B13] Nawrot T. S. Thijs L. Den Hond E. M. Roels H. A. Staessen J. A. (2002). An Epidemiological Re-appraisal of the Association between Blood Pressure and Blood lead: a Meta-Analysis. J. Hum. Hypertens. 16 (2), 123–131. 10.1038/sj.jhh.1001300 11850770

[B14] Needleman H. L. Bellinger D. (1991). The Health Effects of Low Level Exposure to lead. Annu. Rev. Public Health 12, 111–140. 10.1146/annurev.pu.12.050191.000551 1828669

[B15] Nom-011-Ssa1 (1993). Salud ambiental. Limites de plomo y cadmio solubles en artículos de alfarería vidriados. Retrieved from: http://dof.gob.mx/nota_detalle.php?codigo=4801626&fecha=12/11/1993&print=true .

[B16] Nom-047-Ssa1 (2011). Salud ambiental-Indices biológicos de exposición para el personal ocupacionalmente expuesto a sustancias químicas. Retrieved from: http://www.dof.gob.mx/nota_detalle.php?codigo=5249877&fecha=06/06/2012 .

[B17] Nom-147-Semarnat-Ssa1 (2004). Criterios para determinar las concentraciones de remediación de suelos contaminados por arsénico, bario, berilio, cadmio, cromo hexavalente, mercurio, níquel, plata, plomo, selenio, talio y/o vanadio. Retrieved from http://www.dof.gob.mx/nota_detalle.php?codigo=4964569&fecha=02/03/2007.

[B18] Nom-231-Ssa1 (2002). Artículos de alfarería vidriada, cerámica vidriada y porcelana. Límites de plomo y cadmio solubles. Retrieved from https://dof.gob.mx/nota_detalle.php?codigo=5458225&fecha=25/10/2016.

[B19] Nrc (1993). Measuring Lead Exposure in Infants, Children, and Other Sensitive Populations. Washington: National Academy of Sciences. 25144057

[B20] Ntp (2012). NTP Monograph on Health Effects of Low-Level lead, NTP Monogr 23964424

[B21] Nyanza E. C. Dewey D. Bernier F. Manyama M. Hatfield J. Martin J. W. (2019). Validation of Dried Blood Spots for Maternal Biomonitoring of Nonessential Elements in an Artisanal and Small‐Scale Gold Mining Area of Tanzania. Environ. Toxicol. Chem. 38 (6), 1285–1293. 10.1002/etc.4420 30900767

[B22] Pantic I. Tamayo-Ortiz M. Rosa-Parra A. Bautista-Arredondo L. Wright R. Peterson K. (2018). Children's Blood Lead Concentrations from 1988 to 2015 in Mexico City: The Contribution of Lead in Air and Traditional Lead-Glazed Ceramics. Ijerph 15 (10), 2153. 10.3390/ijerph15102153 PMC6210390 30274368

[B23] Resano M. Rello L. García-Ruiz E. Belarra M. A. (2007). Minimally-invasive Filter Paper Test in Combination with Solid Sampling-Graphite Furnace Atomic Absorption Spectrometry for Pb Determination in Whole Blood. J. Anal. At. Spectrom. 22 (10), 9. 10.1039/b706913h

[B24] Rinsky J. L. Higgins S. Angelon-Gaetz K. Hogan D. Lauffer P. Davies M. (2018). Occupational and Take-home Lead Exposure Among Lead Oxide Manufacturing Employees, North Carolina, 2016. Public Health Rep. 133 (6), 700–706. 10.1177/0033354918795442 30231234 PMC6225875

[B25] Rojas‐López M. Santos‐Burgoa C. Ríos C. Hernández‐Avila M. Romieu I. (1994). Use of lead‐glazed Ceramics Is the Main Factor Associated to High lead in Blood Levels in Two Mexican Rural Communities. J. Toxicol. Environ. Health 42 (1), 45–52. 10.1080/15287399409531862 8169996

[B26] Romieu I. Palazuelos E. Hernandez Avila M. Rios C. Muñoz I. Jimenez C. (1994). Sources of lead Exposure in Mexico City. Environ. Health Perspect. 102 (4), 384–389. 10.1289/ehp.94102384 7523102 PMC1566949

[B27] Rothenberg S. J. Schnaas L. Perroni E. Hernandez R. M. Karchmer S. (1998). Secular Trend in Blood lead Levels in a Cohort of Mexico City Children. Arch. Environ. Health Int. J. 53 (3), 231–235. 10.1080/00039899809605700 9814720

[B28] Salud S. d. (2019). Progrma de acción de aplicación Inmediata para el control de la exposición a plomo en México.

[B29] Sanders T. Liu Y. Buchner V. Tchounwou P. B. (2009). Neurotoxic Effects and Biomarkers of lead Exposure: a Review. Rev. Environ. Health 24 (1), 15–45. 10.1515/reveh.2009.24.1.15 19476290 PMC2858639

[B30] Schlenker T. L. Fritz C. J. Mark D. Layde M. Linke G. Murphy A. (1994). Screening for Pediatric Lead Poisoning. JAMA 271 (17), 1346–1348. 10.1001/jama.271.17.134610.1001/jama.1994.03510410058033 8158820

[B31] Shefsky S. (1997). “Comparing Field Portable X-Ray Fluorescence (XRF) to Laboratory Analysis of Heavy Metals in Soil,” in International Symposium of Field Screening Methods for Hazardous Wastes and Toxic Chemicals (NV: Las Vegas). Paper presented at the.

[B32] Shraideh Z. Badran D. Hunaiti A. Battah A. (2018). Association between Occupational lead Exposure and Plasma Levels of Selected Oxidative Stress Related Parameters in Jordanian Automobile Workers. Int. J. Occup. Med. Environ. Health 31 (4), 517–525. 10.13075/ijomeh.1896.01243 29410551

[B33] Silbergeld E. K. Sauk J. Somerman M. Todd A. McNeill F. Fowler B. (1993). Lead in Bone: Storage Site, Exposure Source, and Target Organ. Neurotoxicology 14 (2-3), 225–236. 10.1002/ert.3910200211 8247396

[B34] StataCorp (2017). Stata Statistical Software. College Station, TX: StataCorp LLC.

[B35] Tamayo-Ortiz M. Navia-Antezana J. (2018). Reduced Lead Exposure Following a Sensitization Program in Rural Family Homes Producing Traditional Mexican Ceramics. Ann. Glob. Health 84 (2), 285–291. 10.29024/aogh.916 30873779 PMC6748182

[B36] Tang R. Yang H. Choi J. R. Gong Y. You M. Wen T. (2017). Capillary Blood for point-of-care Testing. Crit. Rev. Clin. Lab. Sci. 54 (5), 294–308. 10.1080/10408363.2017.1343796 28763247

[B37] Téllez-Rojo M. M. Bautista-Arredondo L. F. Richardson V. Estrada-Sánchez D. Ávila-Jiménez L. Ríos C. (2017). Intoxicación por plomo y nivel de marginación en recién nacidos de Morelos, México. Salud Publica Mex 59 (3), 218–226. 10.21149/8045 28902309

[B38] Téllez-Rojo M. M. Bautista-Arredondo L. F. Trejo-Valdivia B. Cantoral A. Estrada-Sánchez D. Kraiem R. (2019). Reporte nacional de niveles de plomo en sangre y uso de barro vidriado en población infantil vulnerable. Salud Publica Mex 61 (6), 787–797. 10.21149/10555 31869543

[B39] Tighe M. Knaub C. Sisk M. Ngai M. Lieberman M. Peaslee G. (2020). Validation of a Screening Kit to Identify Environmental lead Hazards. Environ. Res. 181, 108892. 10.1016/j.envres.2019.108892 31735346

[B40] Who (2010). Exposure To Lead: A Mejor Public Health Concern World Health Organization. Retrieved from https://www.who.int/ipcs/features/lead.pdf?ua=1.

